# Main barriers to effective implementation of stroke care pathways in France: a qualitative study

**DOI:** 10.1186/1472-6963-14-95

**Published:** 2014-02-28

**Authors:** Kristel Gache, Henri Leleu, Gérard Nitenberg, France Woimant, Marie Ferrua, Etienne Minvielle

**Affiliations:** 1 Compaq-HPST, Institut de Cancérologie Gustave Roussy, 114 rue Edouard Vaillant, Villejuif 94805, France; 2 Agence régionale de Santé Ile-de-France, Paris, France

**Keywords:** Stroke, Clinical pathways, Quality of care, Care coordination, Implementation barriers

## Abstract

**Background:**

Stroke Care Pathways (SCPs) aim to improve quality of care by providing better access to stroke units, rehabilitation centres, and home care for dependent patients. The objective of this study was to identify the main barriers to effective implementation of SCPs in France.

**Methods:**

We selected 4 types of SCPs currently implemented in France that differed in terms of geographical location, population size, socio-economic conditions, and available health care facilities. We carried out 51 semi-structured interviews of 44 key health professionals involved in these SCPs and used the interview data to (i) create a typology of the organisational barriers to effective SCP implementation by axial coding, (ii) define barrier contents by vertical coding. The typology was validated by a panel of 15 stroke care professionals.

**Results:**

Four main barriers to effective SCP implementation were identified: lack of resources (31/44 interviewees), coordination problems among staff (14/44) and among facilities (27/44), suboptimal professional and organisational practices (16/44), and inadequate public education about stroke (13/44). Transposition of the findings onto a generic SCP highlighted alternative care options and identified 10 to 17 barriers that could disrupt continuity of care.

**Conclusion:**

Lack of resources was considered to be the chief obstacle to effective SCP implementation. However, the main weakness of existing SCPs was poor communication and cooperation among health professionals and among facilities. We intend to use this knowledge to construct a robust set of quality indicators for use in SCP quality improvement initiatives, in comparisons between SCPs, and in the assessment of the effective implementation of clinical practice guidelines.

## Background

Stroke is a major public health issue worldwide and especially in industrialised countries [1]. Incidence rates range from 73 to 223 cases/100 000 citizens/year according to region [2,3]. The disease is the second, sometimes even leading, cause of death in many countries [4,5]. It is also a major cause of disability and cognitive decline, of worsening of quality of life, and of dependency in one in two patients [6]. In 2008, in France (population, 64.3 million), 105,000 people were admitted to hospital for stroke and 31,000 for transient ischemic attack (TIA), with 24 to 38 stroke-attributable deaths occurring per 100,000 persons per year [7].

Stroke Care Pathways (SCPs) have been set up in North America and several European countries [8-13]. Their general framework embraces multidisciplinary care, care coordination and overall cooperation, and includes the following components: first contact with providers, notification of emergency medical services (EMS) and their response to ensure speedy access to stroke units, acute and sub-acute stroke care, access to rehabilitation and chronic care centres, and provision of home care for dependent patients. Patient access to well-organised, multidisciplinary care in stroke units has been shown to improve quality of service and ultimately to reduce stroke morbidity/mortality rates [14]. Nevertheless, there is little sound evidence for an unambiguous causal relationship between mortality and process measures in stroke care [15]. The development and maintenance of complex cross-boundary SCPs therefore remains an important challenge for clinicians, healthcare managers, and policymakers [16,17]. Fragmented pathways, suboptimal care coordination and poor staff collaboration have hampered the translation of major advances in diagnosis and treatment into clinical practice, resulting in wasted resources and disappointing outcomes [18-20].

The aim of this study was to identify the barriers encountered by healthcare professionals in France when implementing SCPs. Barriers are defined as the causes of discontinuities encountered in daily practice, a continuity being defined as “the delivery of services by different providers in a coherent, logical, and timely fashion” [21]. The study was part of a national project for the development of quality indicators (QIs) for care pathways for use by French regional healthcare agencies. The development of QIs specifically for hospital use requires a review of the literature and of clinical guidelines. The development of QIs for care pathways requires in addition a qualitative analysis of existing care pathways in order to highlight regional differences and reveal the main factors influencing quality of care [22].

## Methods

### Setting

The study was initiated in October 2011 by a healthcare management research team (COMPAQH-HPST, Coordination for Measuring Performance and Assuring Quality in Hospitals – Hospital, Patient, Safety, Territory) whose. The team’s main remit is to develop validated QIs for on behalf of the French healthcare authorities [23]. The task of developing QIs for SCPs was set by the French Ministry of Health and the National Authority for Health (*Haute Autorité de Santé* - HAS) who commissioned the present study and officially exempted us from Ethics Committee approval. Authorisation to conduct such qualitative studies has been granted by the French commission for information technology and civil liberties (“*Commission nationale de l’informatique et des libertés”* -CNIL) which ensures that information technology remains at the service of citizens and does not jeopardise human identity nor breach human rights, privacy or individual or public liberties (Authorisations 1512407v0 and 1518399 v0).

Many care providers are involved in stroke management in France. In each county (*département*), a SAMU (*Service d’Aide Médicale Urgente*) call centre coordinates pre-hospital EMS 24/7. One or more on-duty physicians counsel callers, dispatch the fire brigade, an ambulance or a mobile intensive care unit on site, and enquire into bed availability in hospitals accepting stroke patients [24]. Hospital stroke units manage patients either autonomously or via a telemedicine link with other hospitals. The downstream sector includes rehabilitation centres, nursing or residential care homes, homecare, and general practitioners (GPs). Regional health agencies have a supervisory role with special responsibility for coordination between medical and social services.

### SCP selection

The French regional health agencies designated the members of a 15-strong panel whose overall expertise covered all key aspects of a SCP (hospital managers, neurologists, GPs, a SAMU coordinator, EMS physicians, heads of fire brigades, physiotherapists, coordinating nurses, Physical and Rehabilitation Medicine (PRM) and home care specialists, and patients’ representatives). The panel members derived criteria for discriminating among the various SCP types covered by the general framework described in the introduction by analysing data on inequalities in access to stroke care (data from the National Institute of Statistics and Economic Studies on socio-educative level, social isolation, urban distribution, territorial density of PRM structures [25]; Ministry of Health data on mortality rates in stroke patients and on the national geographical distribution of stroke units). They identified 5 discriminatory criteria: (1) Geographical location (urban, semi-rural, rural (plain or mountain)); (2) Socio-economic and educational conditions; (3) Social isolation of the population and characteristics of the local health care system; (4) Number of stroke units and distance between patient’s home and unit; (5) Availability of neurological or general rehabilitation centres. These 5 criteria, when applied to 6 geographical locations in France differing markedly in surface area, population, number and distribution of stroke units, and organisation of care, revealed 4 SCP types (A to D) (Table 1): (A) large city with good facilities (Paris); (B) urban area with at-risk and isolated population (Seine-Saint-Denis in Ile-de-France); (C) semi-rural or rural areas with distant facilities (Seine-et-Marne and Picardie); (D) isolated mountain or hilly areas (Franche-Comté and Arette vicinity in Aquitaine).

**Table 1:** Characteristics of the 6 areas (a to f) according to the 5 SCP selection criteria leading to the identification of 4 SCP types (A to D)

	**Type of area**	**Socio-economic conditions**	**Social isolation**	**Number of stroke units (distance)**	**Rehabilitation facilities**	**SCP type**
a. Paris	Urban	Good	Low	6 (short)	+++	A
b. Seine-Saint-Denis	Urban	Poor	High	2 (short)	++	B
c. Seine-et-Marne	Semi-rural (plain)	Fair	Average	1 (long)	+	C
d. Picardie	Rural (plain)	Fair	Average	1 (long)	+	C
e. Franche-Comté	Rural (mountain)	Fair	High	1 (long)	+	D
f. Aquitaine(Arette)	Rural (hills)	Fair	Very high	None	None	D

Data from INSEE report, 2013 [25].

### Data collection

**Figure 1:**
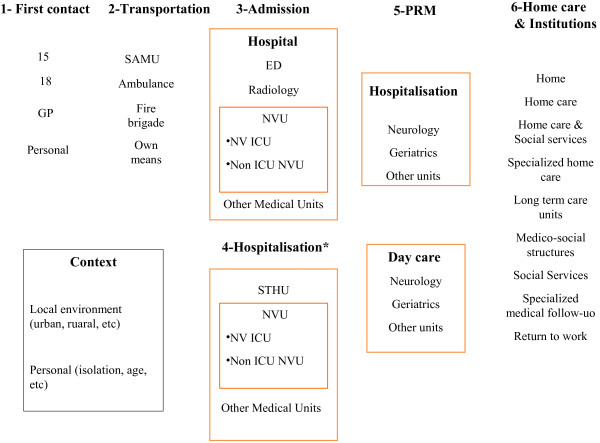
**Grid used for the interview.** GP: General Practitioner; SAMU: *Service d’Aide Médicale Urgente* (coordination of pre-hospital EMS); ED: Emergency Department; NVU: Neurovascular Unit; ICU: Intensive Care Unit; STHU: Short Term Hospitalization Unit. * For inter-hospital transfers, the following information was required: mode of transportation, whether hospitals received a phone call before patient’s arrival, available image transfer facilities, availability of neurologist’s report, and whether thrombolysis had been performed.

Data was obtained from interviews as direct observation of individual patient pathways was not possible. Part of the interviews was based on the analysis of a grid of the main components of a SCP (Figure 1), designed by two researchers (KG, EM) on the basis of official documents classified by level of evidence when available, experiences reported in other areas and countries (e.g. US and UK) [8-10,26] and the advice of the neurologist (FW) in charge of stroke care in Ile-de-France (areas a to c in Table 1). The grid was submitted to the heads of the 3 other regional healthcare agencies (d to f) who, after approving the study, established a list of potential interviewees with expertise in SCPs in their region. These contact persons belonged to the pre-hospital sector (EMS, GPs) or hospital sector (emergency physicians, neurologists, geriatricians, social workers) or were health professionals working in rehabilitation centres, nursing homes for aged dependent persons (*Etablissements d’Hébergement pour Personnes Agées Dépendantes* or *EHPAD*), care homes for the disabled (*Maisons d’Accueil Spécialis*é or *MAS*), and in home care.

The interviews took place between July 2011 and March 2012. Forty-five persons were approached. Final sample size was given by theoretical saturation, i.e. until data from interviews no longer enriched the categorical dimensions of SCPs [27]. The interviewee’s consent was first recorded (one refusal) after which the interview lasted an average of about 30 minutes. Using a semi-structured questionnaire, interviewees were asked to insert arrows between the SCP components in the grid that best described their experience of flow within a SCP, and to indicate where major barriers to continuity of care occurred. They were then asked to comment freely on the content of each barrier. Space was allowed for the emergence of dimensions not included in the initial model. The interview was conducted on a one-to-one basis by either KG or HL, but KG was always present to ensure that interview handling was consistent and that the meaning and relevance of each barrier was as explicit as possible. Overall, 33 interviews were conducted with health professionals from areas a-c and f (see Table 1) (6 pre-hospital, 12 hospital, and 15 post-discharge) followed by 11 interviews with professionals from areas d and e (9 hospital and 2 rehabilitation centre/nursing home staff). Seven additional interviews were conducted by KG to confirm or elaborate on data obtained during the first 33 interviews, bringing the total to 51 interviews of 44 interviewees. The methods used in our study complied with RATS guidelines (see [28]).

### Data analysis

Two researchers (KG, HL) processed and then categorised interview data using axial coding to relate interviewee responses to SCP components and to analyse the extent to which views were shared among interviewees [29]. Using data from the first 33 interviews, the researchers created a typology of barriers which was revised twice, firstly after addition of the data from the next 11 interviews, and then again after addition of the data from the final 7 interviews. Vertical coding was used for a better definition of the meaning of each barrier. Each result was compared to the others in order to determine the sub-dimensions of a given barrier [27,30]. Dependency chains were sought to distinguish barrier-related from clinical practice related data [31]. The content of each barrier was defined after the first 33 interviews and fine-tuned after introduction of the additional data. The typology and barrier contents were discussed and validated first by 4 COMPAQ-HPST researchers (HL, GN, FW, EM) and then by the 15-member expert panel of stroke care professionals. Discrepancies were resolved and minor adjustments were made to the initial model and to the grid during two 3-hour physical sessions.

## Results

### Barrier typology

**Figure 2:**
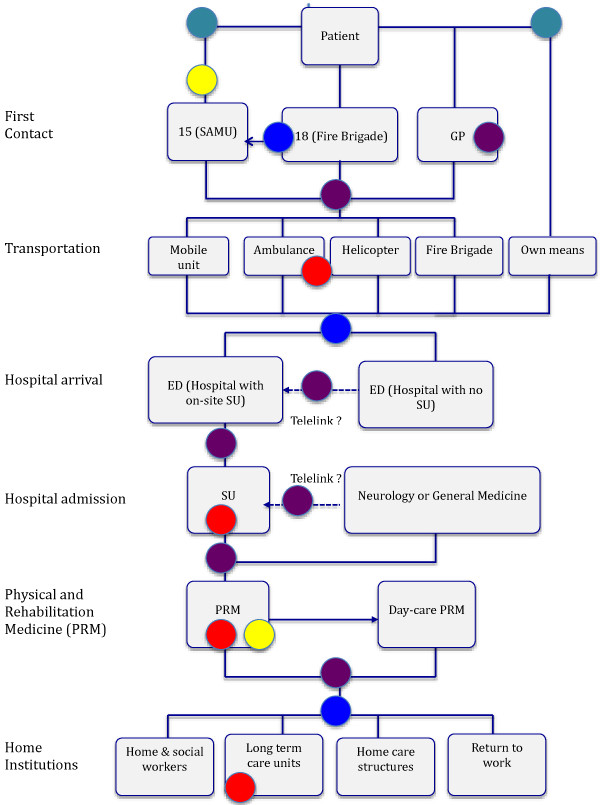
**Journey of an acute stroke patient (generic Stroke Care Pathway) showing the possible barriers leading to discontinuities in care.** Arrows: alternative paths; Coloured disks: barriers leading to discontinuities in care: red disks: lack of resources; brown disks: coordination problems within networks; blue disks: coordination problems between facilities; yellow disks: Problems relative to professional and organisational practices; turquoise disks: public education. GP: General Practitioner; SAMU: *Service d’Aide Médicale Urgente* (coordination of pre-hospital EMS); ED: Emergency Department; NVU: Neurovascular Unit; ICU: Intensive Care Unit; STHU: Short Term Hospitalization Unit.

In general, the interviewees were aware of the evidence provided by the literature on SCPs and were convinced that SCPs promoted patient safety, increased patient satisfaction, and optimised the use of resources. They pinpointed the steps where, according to their own experience, they had encountered technical, cultural or/and organisational barriers when applying recommended principles governing SCP implementation in France [32]. According to SCP type, 10 to 17 barriers that might cause discontinuities of care were identified. These barriers could occur at any step of the general framework, from the emergency call up to admission to an institution or return home or to work (see coloured disks, Figure 2). They were clustered into 4 types with their frequency of occurrence: (i) lack of resources (red disks), (ii) coordination problems within networks (data availability and sharing, information transfer, communication) (brown disks) and between healthcare facilities (blue disks), (iii) problems relative to professional and organisational practices (yellow disks), (iv) public education (turquoise disks) (Table 2).

**Table 2:** Barriers to effective SCP implementation identified by healthcare professionals

**Barrier typology**	**Interviewees n/44 (%)**	**Illustrative examples of verbatims**
Coordination within network: data availability and sharing passing on information staff communication	14 (32)	Waiting times too long when calling the SAMU^1^
		Fire brigade and triage nurses not familiar with stroke symptoms^2^
		Residents not trained to recognise stroke symptoms^3^
		Hospital reports not transmitted to downstream facilities in good time^4^
Coordination between facilities	27 (61)	Disagreements between EMS and neurologists about patient care ^5^
		Hospital physicians unaware of downstream facilities admitting stroke patients^6^
		Inappropriate requests for admission to rehabilitation centres^6^
		What the fire brigade decides is not what the SAMU recommends^7^
		Patients taken to hospital emergency department by the SAMU without prior notification^8^
		Administrative procedures for transferring patients to downstream structures too long^9^
Professional and organisational practices	16 (36)	No established hospital protocol for stroke management^10^
		Patients refused by stroke units in order to keep beds available for patients who are eligible for thrombolysis^11^
		Patients not admitted to rehabilitation centres for financial reasons^12^
Public education	13 (29)	No or little knowledge of stroke symptoms, disease seriousness or treatments^13^
		No knowledge of pre-hospital EMS or how to call them^14^
Logistic resources	31 (70)	No ambulances or helicopters for patient transport^15^
		No beds available in stroke units^16^

SAMU: *Service d’Aide Médicale Urgente* (coordination of pre-hospital EMS);

EMS: Emergency Medical Services.

Origin of the verbatims

^1^GP; ^2^EMS specialist; ^3^Neurologist; ^4^Neurologist and PRM physician; ^5^Neurologist; ^6^Heads of PRM structures; ^7^SAMU coordinator; ^8^Senior resident working in emergency department; ^9^Head of Neurovascular Unit; ^10^Neurologists; ^11^GP and emergency department physician; ^12^Nurse and social worker; ^13^GP and neurologist; ^14^SAMU coordinator, GP and neurologist; ^15^GP in mountain area; ^16^GPs and EMS specialists.

### Lack of resources (red disks)

Lack of resources was the most commonly cited barrier (cited by 31/44 interviewees). The main complaint was staff shortages in rehabilitation centres and nursing homes (n = 28). However, other grievances were unavailability of inpatient beds in stroke units (n = 7) and inadequate logistic resources in pre-hospital care (ambulance and helicopter availability) (n = 6).

### Coordination problems

#### Coordination problems within networks (brown disks)

Coordination problems within networks were mentioned by 14/44 interviewees with misunderstandings often cropping up: (i) between hospital staff and heads of downstream facilities with regard to admission to rehabilitation centres. Physicians did not know which facilities accepted stroke patients and underestimated psychological disorders as well as the need for home care after early discharge; (ii) between GPs and hospital consultants. Consultants complained that GPs often referred patients for tests in the private sector without enquiring about prior hospital admissions for stroke and that they modified prescriptions without seeking advice (e.g. antiplatelet treatment discontinued before next hospital visit); (iii) between specialists in different disciplines, especially between emergency physicians and neurologists.

Information transmission was considered suboptimal, with medical reports being transmitted late to downstream facilities and containing insufficient information on cognitive assessments.

Inadequate training was an issue. Triage nurses, hospital residents and the fire brigade were considered to be inadequately trained in the recognition of stroke symptoms. GPs were considered to have insufficient knowledge of recent advances in stroke medicine, including the extended time window for intravenous tissue plasminogen activator [33].

#### Coordination problems between facilities *(blue disks)*

Coordination problems between facilities were mentioned by 27/44 interviewees. They arose: (i) in the pre-hospital setting between the fire brigade and SAMU in relation to patient orientation; (ii) between the pre-hospital and hospital settings when the SAMU transported patients to a hospital without advance warning and without obtaining a green light; (iii) between the hospital and downstream facilities because of differences in operational schedules. Stroke units operate on a daily basis whereas rehabilitation centre admission committees usually meet once weekly. In addition, administrative procedures took far too long, e.g. recognition of disabled worker status authorising admission to a *Maison Départementale des Personnes Handicapées* or *MDPH*.

### Problems relative to professional and organisational practices (yellow disks)

The interviewees (16/44) pointed out the following problems: overlong SAMU call waiting times; no formal hospital protocol (critical pathway) for stroke care; stroke units keeping beds for thrombolysis patients and refusing patients ineligible for thrombolysis or TIA patients; long wait for examinations (e.g. echocardiography) holding up discharge; patients with financial difficulties refused admission to rehabilitation centres.

### Public education (turquoise disks)

The need for more public education was underscored by the interviewees (13/44), in particular with regard to symptoms, disease seriousness, and treatments (thrombolysis). Too few patients or GPs made direct calls to the SAMU.

## Discussion

Our study has identified barriers that health professionals consider to be major obstacles to effective implementation of a SCP. These barriers were identified by analysis of 4 SCP types and related to three main quality concerns: lack of resources, lack of training and public information, and lack of coordination between staff or healthcare facilities. Problems relating to best clinical practices, as translated into evidence-based protocols, were not considered.

The barrier most often mentioned was lack of resources, confirming that France needs to invest more in stroke management despite noteworthy results in some parts of the country [34,35]. The objectives of the ongoing 2010–2014 national Stroke Plan are not only to increase the number of stroke units and to introduce coordinators to increase SCP efficiency, but also to improve training and public information, and to extend stroke care beyond prevention and primary care by setting up a coordinated set of programmes and actions for social care [32]. Between 2010 and 2030, stroke prevalence is estimated to increase by 25% and direct medical treatment costs for stroke care to triple [8]. However, in the current political and economic climate, it is unlikely that more cash will be forthcoming for public health spending. Continuity of care will therefore probably decline as resources dwindle (fewer primary care physicians, more part-time physicians, and greater divisions between hospital consultants and GPs). These shortcomings will need to be offset by better organisation of care.

The interviewees considered that patients and public should have better knowledge of stroke symptoms, and that triage nurses and young residents should be better versed in stroke care in general. Information campaigns on the early recognition of stroke symptoms and on the seriousness of the disease need to be set up nationally, regionally, and for special population subgroups (e.g. relatives of acute stroke patients) [36], despite the current lack of evidence for the effectiveness of such interventions [37]. QIs developed for regional health agency use should take account of these knowledge and information needs, and should be developed from a holistic perspective that covers personal risk factors for stroke, warning signs of stroke, EMS activation, need for follow-up after discharge, and medications management, and could also include the consumer perspective [38] and patient experience [39].

The third barrier to be identified was poor coordination between staff and between healthcare facilities. The issue of poor coordination has already been raised by physicians [26,40,41] and has been shown to impact on both quality and costs [42]. Reported examples of poor coordination are lack of treatment follow-up, conflicting information from providers, delayed information transfer following hospital discharge, and unavailability of relevant information during patients’ scheduled visits. Care coordination is increasingly seen as an evidence-based aspect of high-quality health care delivery but needs to be integrated both horizontally (peer-based and cross-sector collaboration) and vertically (patient pathways transcending organisational boundaries) in order to result in healthcare benefits [43]. There is, however, no consensus about what constitutes care coordination, how to build an evidence base for care coordination [44], and how to develop coordination measures [45].

We shall consider coordination within the pre-hospital setting, hospital setting, and post-hospital setting in turn. In the pre-hospital setting where “time is brain”, our interviewees considered that closer collaboration was required between pre-hospital EMS and hospitals for prompt patient transfer to a facility with appropriate resources. This is an evidence-based measure that has been underscored by the Stroke Systems Work Group in California [9] and by the Madrid acute stroke care programme [12]. Admission delays have already been considerably reduced in a regional network in France by close collaboration with pre-hospital EMS [46]. However, an outstanding problem that the French regional health agencies will need to solve is that the fire brigade, when called out by the patient or by the GP, will not transport a patient to a hospital outside its jurisdiction area. This causes misunderstandings and conflicts between the fire brigade and SAMU. The interviewees also complained of lack of communication and cooperation within the hospital setting, with emergency physicians and neurologists often in disagreement over what was appropriate care and with too little liaising among staff (e.g. between emergency physicians, radiologists, laboratory personnel, neurologists). Proposed corrective actions are team-working, implementation of critical pathways in stroke units [12,41,47,48], recognition of the key role played by nurses [49], and the setting up of stroke teams in hospitals with no stroke unit or with scant resources [50]. Coordination with EMS has led to more widespread use of thrombolytic therapy [24]. The main coordination problems encountered after patient discharge from hospital were poor information transfer from hospital to downstream staff, on the one hand, and delays due to administrative procedures, on the other [51]. Solutions to such problems might be holistic initiatives such as, for instance, integrated PRM care pathways [6,52] and early supported discharge procedures which have been shown to reduce long-term dependency, admissions to institutional care facilities, and length of hospital stay, at least in a selected elderly group of stroke patients with moderate disability [53]. In summary, for a SCP to function well, health professionals need to collaborate closely. The introduction in France of SCP coordinators involved in setting up and running SCPs may help promote collaboration.

The barriers to effective SCP implementation depended on geographical location (urban/rural (mountains or plains), distance to stroke unit) and patient background (isolation, socio-economic and educational status). Inequalities in access to healthcare by area of residence have already been highlighted [8,9,26]. In semi-rural areas around Paris (e.g. Seine et Marne), the stroke centre may be as far as 80 miles away. Air transportation has thus to be developed to ensure equitable access for all. Improvement initiatives and their costs will vary according to the healthcare system and the facilities available within the system [42]. Discrepancies might be reduced and higher quality care might be provided by adopting initiatives developed in other large rural areas (e.g. Alberta’s Provincial Stroke Strategy and Telestroke program) [13].

Our study has limitations: (i) it is not an exhaustive overview of the implementation of SCPs as the interviewees did not mention some key evidence-based measures facilitating implementation, no doubt because they had not experienced these barriers. Such crucial “facilitators” include the importance of “champions” or “fixers” for successful policy implementation [13,54], the benefit of establishing clear, relevant, flexible policies to motivate and focus stakeholders on key issues [9,10], and the positive role of health care competition and incentives (not restricted to financial ones) to facilitate a wider adoption of policies and to promote improved quality of care [29,55]; (ii) our study relied on interview data and documentation and not on real-world observations. Observations during follow-up of patients with stroke are particularly difficult, especially in emergent situations; (iii) data provided by 44 volunteer interviewees from just four regions of France cannot provide a picture of the overall national context; (iv) we did not interview patients or their next of kin about their experiences or for their views on an ideal SCP; (v) our findings apply to the French healthcare system and may not be more widely applicable even though they are often supported by findings obtained elsewhere.

Our findings have practical implications for the development of QIs for SCPs for use by French regional healthcare agencies: (i) Improvement initiatives will have to focus on flaws in organisational aspects, especially on coordination between staff and between facilities, as the major barriers identified concerned organisational issues rather than best clinical practices [44]. Developing QIs for networks, in which often competing facilities have to cooperate, is more complex than developing QIs for a single facility. QIs for networks relate to the dynamics of a social practice rather than to reified procedures and standards, and have to confer visibility to routine coordinating actions [56]; (ii) More than one type of SCP will need to be validated as SCPs depend on local geography but the number should nevertheless remain limited [57]; (iii) QIs for assessing SCP quality in different settings will have to be metrologically validated. Preliminary work has been carried out at the European level [58,59] and in France for the referral of patients from acute to rehabilitation care [60]; (iv) Recent experiences support the assumption that well-organised, multidisciplinary care can improve quality of service and reduce stroke-related mortality and morbidity rates [40,61] for acute stroke [14,15,41,62] as well as for stroke rehabilitation [18].

## Conclusions

Barriers corresponding to discontinuities of care within 4 types of SCP were identified during interviews of healthcare professionals. The main weakness of SCPs was poor communication and cooperation among health professionals and among facilities as providers of a continuum of effective care. We intend to apply this newly acquired knowledge and our existing expertise in QI development [63] to construct a robust set of process QIs [64], and ultimately outcome QIs, for use in SCP quality improvement initiatives. Each regional health agency will be able to use a composite QI to compare its SCPs, to analyse results according to population criteria (socio-economic status, social isolation), geographic criteria (location, distance) and available facilities (number of stroke units and rehabilitation centres), and assess quality of stroke care in general “in the context of the current health care bureaucracy and economic reality” [65].

## Competing interests

The authors declare they have no competing interests.

## Authors’ contributions

KG made substantial contributions to design of the study, participated in the acquisition of data and was involved in the drafting of the manuscript. HL and MF participated in the acquisition of data and in discussions on data interpretation. GN was involved in the conception, design, and vetting of the study and in the drafting of the manuscript. FW and EM oversaw the work (FW from the viewpoint of the regional healthcare agencies and EM from that of overall healthcare management) and critically revised the manuscript. All authors read and approved the final manuscript.

## Pre-publication history

The pre-publication history for this paper can be accessed here:

http://www.biomedcentral.com/1472-6963/14/95/prepub
